# Food Agency in the United States: Associations with Cooking Behavior and Dietary Intake

**DOI:** 10.3390/nu12030877

**Published:** 2020-03-24

**Authors:** Julia A. Wolfson, Jacob Lahne, Minakshi Raj, Noura Insolera, Fiona Lavelle, Moira Dean

**Affiliations:** 1 Department of Health Management and Policy, University of Michigan School of Public Health, Ann Arbor, MI 48109, USA; miraj@umich.edu; 2 Department of Nutritional Sciences, University of Michigan School of Public Health, Ann Arbor, MI 48109, USA; 3 Virginia Tech, Blacksburg, VA 24061, USA; jlahne@vt.edu; 4 Institute for Social Research, University of Michigan, Ann Arbor, MI 48104, USA; nehamid@umich.edu; 5 Institute for Global Food Security, School of Biological Sciences, Queen’s University Belfast, Belfast BT9 5DL, UK; flavelle01@qub.ac.uk (F.L.); moira.dean@qub.ac.uk (M.D.); 6 School of Health Sciences, Faculty of Health and Medicine, The University of Newcastle, Callaghan, NSW 2308, Australia

**Keywords:** food agency, cooking, dietary intake, diet quality, survey, cooking skills, food skills

## Abstract

“Food agency” is one’s ability to procure and prepare food within the contexts of one’s social, physical, and economic environment. In 2018, we used Amazon TurkPrime to field two large national surveys in the United States (US) to examine food agency and several food- and cooking-related factors. The first survey (*n* = 1,457) was fielded in a national sample of US adults. The second survey (*n* = 1,399) comprised of parents of 2–9-year-old children. Analyses included hierarchical linear regression to examine factors that explained variation in food agency and used Poisson and generalized linear models to examine the association between food agency and between cooking behavior and dietary intake, respectively. Cooking skills; food skills; and cooking confidence, attitudes, and perceptions explained a high degree of food agency variance. Higher food agency was associated with more frequent cooking of all meals, more frequent scratch cooking, and less frequent cooking with packaged ingredients among both adults and parents. Higher food agency was also associated with higher consumption of vegetables among both adults and children. Food agency encompasses a number of the interrelated factors important for home cooking and is a useful construct for understanding and promoting home cooking behavior.

## 1. Introduction

Cooking meals at home is a key recommendation for consumption of a healthy diet [1], and evidence shows cooking frequently is associated with better diet quality and higher fruit and vegetable consumption [2,3,4,5,6]. The public health focus on cooking as an important health behavior has emerged in response to high rates of diet-related diseases such as obesity, diabetes, and hypertension and strong evidence about the effects of fast foods and ultra-processed foods on those outcomes [7,8,9,10]. Numerous interventions focus on promoting cooking skills and healthy cooking at home in the United States (US), where cooking skills are perceived to have declined [11]. However, evidence regarding the effectiveness of cooking interventions on improving skills, cooking behavior, and diet and health outcomes is limited [11]. “Food agency” is a construct that positions the act of cooking within the myriad factors that influence one’s ability (a) to obtain cooking skills and (b) to translate those skills into action within the contexts of one’s social, physical, and economic environments [12,13]. Food agency is, therefore, a useful lens for capturing and understanding cooking skills and behavior and how interventions can best promote healthy cooking behavior that reflects the reality of the constraints of daily life.

Cooking—assembling and transforming ingredients in order to produce a meal—is a complex behavior that requires numerous skills and competencies that relate to the technical act of preparing food and to the ability to plan to cook and provision food within one’s food environment, time constraints, budget constraints, and so on [13,14,15]. Cooking also has neither clear definition nor one “correct” set of actions, and the understanding of what it means “to cook” is complicated by the wide range of processed and packaged ingredients and food products available in stores [16,17,18,19]. The complicated reality of what is required to cook within one’s daily life challenges prescriptive conceptualizations of how cooking skills have been previously understood and measured [16,20]. Cooking skills have sometimes been measured as the ability to prepare certain sauces or dishes or the ability to use certain cooking techniques [21]. More recently, scales to measure cooking skills as well as related food skills have been developed [22]. These scales have expanded the number and types of skills considered important for home cooking and have shown that both cooking and food skills are critical for shaping dietary intake [23]. Other measures of cooking confidence and attitudes as well as how people perceive what it means to cook (related to the role of convenience foods vs. scratch ingredients and whether heat is required for cooking) have also been shown to be important predictors of cooking behavior [24,25,26,27]. 

The Cooking and Food Provisioning Action Scale (CAFPAS) is a 28-item scale developed to measure food agency [28]. However, to our knowledge, it has neither been tested in a large national sample nor been fielded among a sample of parents specifically. Cooking skills and practices can change throughout the life course with parenthood being a particularly pivotal point due to the importance of a providing a healthy diet for child dependents [29,30,31,32]. Numerous cooking and nutrition interventions are aimed at parents and at parents of young children specifically, as evidence suggests that parental cooking skills and confidence are associated with better diet quality for children [33]. However, parenthood is also an especially busy and stressful time with many demands placed on parents that make preparing healthy, home-cooked meals difficult. It is therefore unknown whether food agency might be higher or lower among parents of young children compared to adults more generally. More detailed knowledge about food agency across the life course would be helpful in the development of targeted cooking and nutrition interventions.

In this study, we sought to better understand how food agency is related to other measures of cooking skills; food skills; and cooking-related confidence, attitudes, and perceptions among parents of 2–9-year-old children and among a general sample of US adults. Food agency is not based on how competent a person is at a predetermined set of skills or actions; rather, having food agency means being “empowered to act” to achieve food-related goals in the contexts of the organizing structures of one’s life [12]. A deeper discussion of the theoretical underpinnings of food agency is available elsewhere [12,13,28]. Despite this intentionally broad conceptualization, it is likely that food agency is closely related to self-perceived cooking and food skills, confidence, attitudes, and perceptions about cooking. We hypothesized that cooking and food skills would explain a high degree of variation in food agency. A second aim of this research was to examine how food agency is associated with differences in cooking behavior and dietary intake. We hypothesized that, for both adults and parents, higher food agency would be associated with more frequent cooking, particularly cooking from scratch, and would also be associated with better diet quality.

## 2. Materials and Methods

A web-based survey to measure cooking skills, food skills, food agency, cooking attitudes, perceptions about the meaning of cooking, cooking confidence, cooking behavior, and dietary intake was designed. All cooking-related scales and measures were taken from previously validated surveys [22,24,28]. Because the cooking skills and food skills measures had not been validated in a US sample, we conducted “think aloud” cognitive interviews (*n* = 10) to ensure they were understood as intended and adjusted any wording for a US audience. Two versions of the same survey were drafted: Version 1 to be fielded in a sample of US adults (hereafter referred to as the “general adult” survey) and Version 2 (hereafter referred to as the “parent” survey) to be fielded in a sample of parents of at least one 2–9-year-old child in the US. The only difference between the two surveys was that the dietary intake module asked about the adults’ own dietary intake in the general adult survey, and in the parent survey dietary intake, questions referred to the intake of a reference child between the ages of 2–9 years old. 

The surveys were fielded in April 2018 using TurkPrime, an online crowdsourcing platform associated with Amazon Mechanical Turk that is designed to be used for academic research [34]. Amazon Mechanical Turk and TurkPrime have been used in numerous academic studies from a variety of disciplines published in the peer-reviewed literature [35,36,37]. TurkPrime is a panel service that allows researchers to use quotas to recruit a sample that matches their specific needs. In the case of the present study, we used a census-matched panel that generated a final sample proportionally matched to the US adult population based on prespecified characteristics. For the general adult survey, the sample proportionally matched the US adult population based on age, sex, race/ethnicity, education, and income. For the parent survey, the sample proportionally matched the US adult population based on race/ethnicity, education, and income. 

A pool of 1731 TurkPrime panel members (aged ≥ 18 years) began the general adult survey. Individuals were excluded if they did not finish the survey (*n* = 180), completed the survey in <7 minutes (*n* = 61) as it was not reasonable to complete the survey having read all the questions in that time, or if they did not live in the US (*n* = 33). In total, 274 respondents were excluded, resulting in a final sample size of 1457 and a survey completion rate of 84.2%. A pool of 1756 TurkPrime panel members (aged ≥ 18 years) began the parent survey. Individuals were excluded if they did not finish the survey (*n* = 270), completed the survey in <7 minutes (*n* = 53), did not live in the US (*n* = 4), did not have a child aged 2–9 years (*n* = 13), or had >6 children (*n* = 17). In total, 357 individuals were excluded, resulting in a final sample size of 1399 and a survey completion rate of 79.7%. 

### 2.1. Measures

*Food agency* [12,13] was measured using the Cooking and Food Provisioning Action Scale (CAFPAS) [28]. The CAFPAS is a 28-item scale that asks participants how strongly they agree, on a 7-point Likert scale from “strongly agree” to “strongly disagree”, with statements about food and cooking self-efficacy, attitudes, and structural barriers to cooking. The CAFPAS has three subscales: food and cooking self-efficacy, food and cooking related attitudes, and structural barriers. Item scores within each subscale are standardized, and then, the standardized subscale scores are summed to generate an overall CAFPAS score. We used the continuous CAFPAS scores and also divided scores into quartiles for some analyses. 

*Cooking skills* were measured using a 14-item scale that asked participants “On a scale of 1 to 7, where 1 means very poor and 7 means very good, please say how good you are at …” with response items focusing on specific cooking skills such as steaming, blending, and roasting foods. *Food skills* were measured using 19 items measured on the same 7-point Likert scale; however, in this case, skills focused on skills such as meal planning, shopping with a grocery list, and comparing prices when food shopping. More details about the cooking and food skills measures are published elsewhere [22,23]. 

*Cooking confidence* was assessed by asking participants to rate how confident they were on a 7-point Likert scale from 1 (“not at all confident”) to 7 (“extremely confident”) cooking from scratch using fresh ingredients, following a recipe, cooking a healthy meal, and preparing a meal using a vegetable they have never used before. *Cooking attitudes* were assessed using 12 questions that asked participants to rate how strongly they agreed (on a 7-point Likert scale) with statements such as “I enjoy cooking” and “cooking takes too much time” [24].

*Perceptions about what counts as cooking* were based on responses to 14 items that asked respondents to rate how strongly they agreed or disagreed (on a 7-point Likert scale) with statements that parsed how strongly they thought that packaged, convenience foods, scratch ingredients, and with or without heat counted as cooking. The cooking perception scale is described in detail elsewhere [24].

The order in which participants viewed items for the CAFPAS, cooking skills, food skills, cooking confidence, cooking attitudes, and cooking perceptions scales were randomized. The order in which participant viewed each scale did not differ between participants. 

*Cooking behavior* was measured using seven questions about cooking frequency of breakfast, lunch, and dinner; making a meal from scratch ingredients; making a meal from packaged products; making a meal from frozen products; and using a recipe to make a meal. Participants were asked to “indicate how many times during the past 7 days you or someone in your household did the following…” Response categories ranged from 1 to 7. This question stem was based on the cooking frequency question in the National Health and Nutrition Examination Survey [38]. The full set of cooking behavior questions have also been fielded previously [24]. 

*Dietary intake* was measured based on eight questions from the Behavioral Risk Factor Surveillance System [39]. Questions asked about frequency of eating/drinking each food/beverage in the past 30 days, and participants were asked to report frequency as either times per day, times per week, or times per month. All responses were converted into daily intake by multiplying “per week” responses by 7 and “per month” responses by 30. The full question text for the dietary intake measures are available in the Supplementary Materials. Dietary intake was measured for the surveyed adult in the general adult survey. In the parent survey, the respondent answered the dietary intake questions about the intake of a reference child in their family between the ages of 2 and 9 years old. 

*Sociodemographic measures* included in both surveys were sex, age (18–29, 30–44, 45–64, and ≥ 65), race/ethnicity (non-Hispanic White, non-Hispanic Black, Hispanic, and other), household size (1–4 and ≥5), parent status, number of children in the household, ages of children (0–5, 6–11, and 12–19), marital status (married, living with a partner, divorced/separated/widowed, and never married), annual household income (<$50,000, $50,000–$100,000, and >$100,000), education (≤ high school diploma, some college, college, or graduate degree), employment status (working full time, working part time, not working but looking for work, not working and not looking for work, and stay at home parent), Supplemental Nutrition Assistance Program (SNAP) participation, Special Supplemental Program for Women, Infants and Children Program (WIC) participation, household food insecurity status, and weight status (healthy, overweight, and obese). Adult weight status was calculated based on Body Mass Index (BMI) score calculated based on self-reported height and weight. Healthy weight status = 18.5 < BMI < 24.99, overweight = 25.00 ≤ BMI < 30.00, and obese weight status = BMI ≥ 30. Food insecurity was based on a single question asking “which of the following statements best describes the situation in your household in the last month.” Response categories included the following: we have enough of the foods we want to eat, we have enough but not always the kinds of food we want to eat, sometimes we do not have enough to eat, and we often do not have enough to eat. In the parent survey, the age and sex of a reference child was also recorded. 

### 2.2. Analysis

All analyses were conducted separately for the general adult sample, the parent sample, and the combined sample from the two surveys (except for those examining dietary intake outcomes, which did not combine the adult and parent samples). First, we used descriptive statistics to examine demographic differences in the study samples and the mean scores and distribution of the food agency and cooking skills, attitudes, confidence, and perception measures for each of the study samples. Next, we conducted simple linear regressions with post-estimation margins to examine the unadjusted differences in food agency based on key sociodemographic characteristics. We then conducted hierarchical linear regressions adjusted for the covariates described above to examine the factors that contribute to variation in food agency. Then, differences in cooking behavior and dietary intake based on quartiles of CAFPAS score were examined. Cooking behavior outcomes used Poisson models adjusted for sex, age, race/ethnicity, number of children, income, education, food security, and cooking perceptions. Dietary intake outcomes used generalized linear models with a gamma family and log link to account for the skewed distribution of the dietary intake variables. These models were adjusted for sex, age, race/ethnicity, number of children, income, education, and food security. Models for the parent sample were additionally adjusted for reference child age and sex. Post-estimation margins were used to generate predicted cooking behavior and dietary intake based on quartile of CAFPAS score. All tests were two-sided, and significance was considered at *p* < 0.05. Analyses were conducted with Stata, Version 15 in 2019 and 2020. 

## 3. Results

The characteristics of the two study samples as well as the combined sample are described in Table 1. The general adult sample was evenly divided between males (49.3%) and females (50.7%), whereas the parent sample had a higher proportion of female respondents (65.5%). The mean age of the general adult sample (46.5 (±15.8) years) was older than the parent sample (35.5 years (SD 8.5)). In the general adult sample, 56.2% of the sample were parents, though the mean number of children in the household was lower (1.3 children (SD 1.5)) compared to the mean number of children in the household in the parent sample (2.3 children (SD 1.1)). There were some differences between the distributions of the two samples based on marital status, household size, employment, and SNAP and WIC participation. The samples were similar in terms of race/ethnicity, household income, education, household food security, and weight status.

Table 2 displays the mean scores and standard deviations for the food agency and the cooking skills, confidence, attitudes, and perceptions measures used in the study. Across all measures, mean scores were similar between the general adult and parent samples. Within each sample, however, there were significant differences in food agency based on key sociodemographic characteristics (Table 3). In the general adult sample, food agency was higher among females (compared to males, *p* < 0.001), with increasing age (*p* < 0.001), among nonparents (*p* < 0.001), and among individuals not working and not looking for work or among stay at home parents (compared to individuals working full time, *p* < 0.001). Among the parent sample, food agency was higher among females (compared to males, *p* = 0.002), individuals aged 45–65 years old (compared to 18–29 years old, *p* = 0.03), individuals with a college or graduate degree (compared to individuals with a high school diploma or less, *p* < 0.001), and individuals not working and not looking for work or stay at home parents (compared to individuals working full time, *p* < 0.001). In both groups, lower food security was associated with significantly lower food agency. 

Table 4 presents results from the hierarchical linear regression models testing the relative explanatory contribution of various factors to differences in food agency in the combined sample. Parallel results from the general adult sample and parent sample are shown in Supplementary Materials Table S1 and S2. Model 1, controlling for demographic characteristics sex, age, race, and number of children in the household, explained 4.4% of the variation in food agency. The magnitude, direction, and significance of these variables remained with the addition of socioeconomic measures for household income, education, and household food security in Model 2. These additional variables increased the model R^2^ by 2.1%. Higher income and education were both associated with lower food agency, and this relationship persisted in the next two models. Cooking skills and food skills were added in Model 3 which significantly increased the R^2^ to 41.5% (*p* < 0.001). Both cooking skills and food skills were associated with higher food agency (β = 0.04, *p* < 0.001). Cooking confidence, positive and negative attitudes, and cooking perceptions were added in Model 4. Model 4 explained 71.3% of the variation in food agency (additional variance explained according to R^2^ from Model 3: 29.8%, *p* < 0.001). Cooking skills and food skills remained significant at *p* < 0.001 in Model 4, but the magnitude of their association was attenuated. Cooking confidence (β = 0.15), positive (β = 0.65) and negative (β = -0.73) attitudes, and the three cooking perception factors (convenience foods (β = −0.12), scratch ingredients (β = 0.19), and heat (β = −0.11)) were all significantly associated with food agency at *p* < 0.001. In the final model, sex, age, income, education, and food security remained significant whereas race/ethnicity and number of children in the home were no longer significantly associated with food agency.

**Figure 1:**
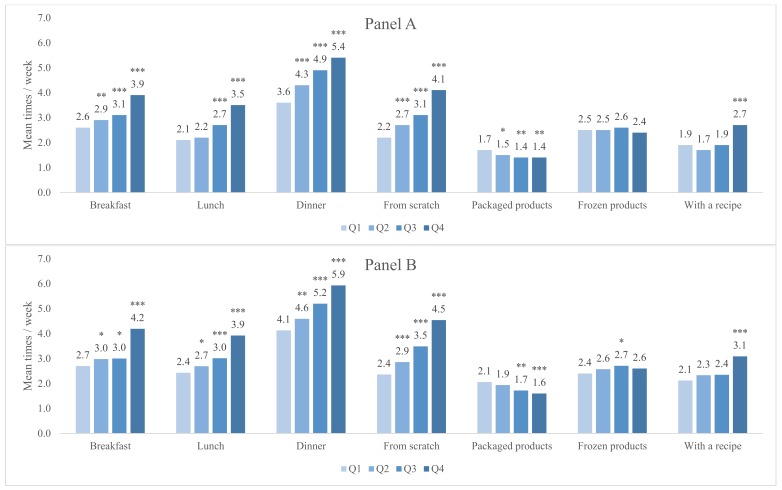
Associations between food agency and cooking behavior among the general adult population (**A**) and among parents of 2–9-year-old children (**B**). Note: Based on poisson regressions adjusted for sex, age, race/ethnicity, number of children, ages of children, income, education, food security status, and cooking perceptions. Parent sample models were further adjusted for the reference child age and sex. Frequency of cooking behaviors is measured over the past 7 days. Difference from Q1 significant at *** *p* < 0.001, ** *p* < 0.01, and * *p* < 0.05.

Associations between quartiles of food agency and cooking behavior among the general adult population (Figure 1A) and among parents specifically (Figure 1B) are shown in Figure 1. Among both adults and parents, higher food agency was associated with greater frequency of cooking breakfast, lunch, and dinner (*p* < 0.001 for Q4 vs. Q1 for each meal in both samples). Higher food agency was also associated with more frequent cooking from scratch (*p* < 0.001 for Q4 vs. Q1 for both samples) and less frequent cooking with packaged products (*p* < 0.001 for Q4 vs. Q1 for both samples). Supplementary Figure 1 shows the associations between food agency and cooking behavior in the combined sample. 

**Figure 2:**
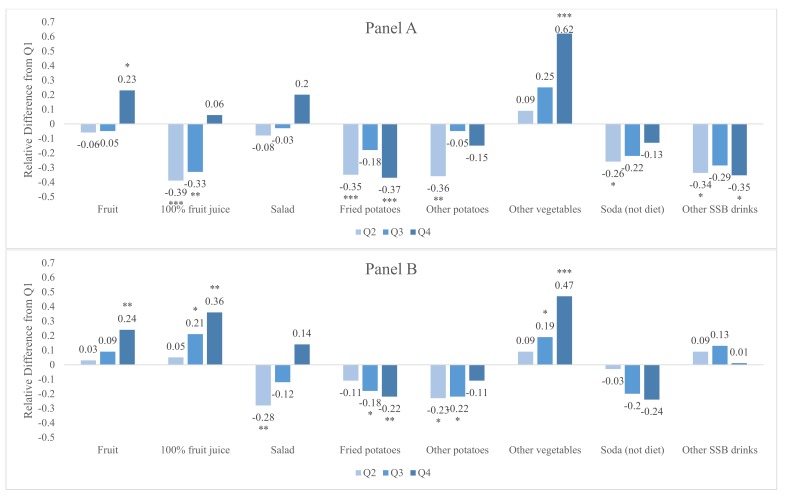
Associations between food agency and dietary intake among the general adult population (**A**) and parental food agency and dietary intake of 2–9-year-old children (**B**). Note: Based on generalized linear models with log link and gamma family adjusted for age, sex, race/ethnicity, number of children, ages of children, income, education, and food security status. Difference from Q1 significant at *** *p* < 0.001, ** *p* < 0.01, and * *p* < 0.05.

Figure 2 shows the associations between food agency and dietary intake among adults (Figure 2A) and the associations between parental food agency and child dietary intake (Figure 2B). Among the general adult population, higher food agency was associated with higher fruit and other vegetables daily intake (Q4 vs. Q1: fruit, *p* = 0.02; other vegetables, *p* < 0.001). Higher food agency was also associated with lower consumption of 100% fruit juice (Q2 vs. Q1: *p* < 0.001), fried potatoes (Q4 vs. Q1: *p* < 0.001), other potatoes (Q2 vs: Q1: *p* = 0.001), soda (Q2 vs. Q1: *p* = 0.04), and other sugar sweetened beverages (Q4 vs. Q1: *p* = 0.02). Higher food agency among parents was associated with high fruit (Q4 vs. Q1: *p* = 0.02), 100% fruit juice (Q4 vs. Q1: *p* < 0.001), and other vegetable intake (Q4 vs. Q1: *p* < 0.001) among their 2–9-year-old children. Higher parental food agency was also associated with less consumption of salad (Q2 vs. Q1: *p* = 0.002), fried potatoes (Q4 vs. Q1: *p* = 0.007), and other potatoes (Q2 vs. Q1: *p* = 0.02) among 2–9-year-old children. 

## 4. Discussion

To our knowledge, this is the first study to examine food agency and the complex factors associated with it in a large, national sample in the US. We find that differences in cooking skills, food skills, cooking confidence, and attitudes and perceptions about cooking rather than demographic or socioeconomic factors explain most of the variation in food agency among US adults. We also find that higher food agency is associated with clear differences in cooking behavior and dietary intake in ways that may be important for improving diet quality and diet-related health outcomes among both adults and children. One reason for this may be that cooking is a complex activity that must take place within the parameters of one’s daily life. Put another way, simply having cooking skills is not sufficient; one must be able to use them and to use them frequently. Therefore, food agency (and the CAFPAS scale to measure it) may be a valuable construct that effectively and succinctly summarizes disparate and nonoverlapping cooking and food constructs and skills in the prediction of cooking related outcomes. 

The present findings regarding food agency are generally consistent with prior literature examining demographic differences in cooking and food skills, particularly our findings regarding higher food agency among females as compared to males, and differences based on age, education, and employment status [21,23,40,41]. We did not observe notable differences in food agency based on race/ethnicity. Interestingly, in this study, we also observed higher food agency scores among parents compared to adults more generally. Among parents of 2–9-year-old children, univariate analyses indicate that food agency was lower among parents with a college or graduate degree compared to those with a high school diploma or less. In both the parent and adult samples, food agency was lower among those working full time compared to stay at home parents or those not working and looking for work. These differences were driven by significantly lower scores in all three subscales with the largest difference found in the structure subscale. This is a notable finding as it speaks to the fact that cooking skills and behavior do not occur in a vacuum and that one’s ability to put them into practice in daily life—in other words to have agency—is dependent on other contextual factors including having time available to cook, work schedules, and responsibilities [12,13,15,20]. 

Results from the multivariable hierarchical regression models add further complexity to current understanding of the factors that influence food agency [12,13,28]. As we would predict, demographic and socioeconomic factors alone explained very little of the variation in food agency, whereas the addition of cooking skills and food skills measures to the model increased the R^2^ from 6.4% to 41.5%. The addition of cooking confidence, attitude, and perception measures also resulted in a significant increase in the amount of variance explained (final model R^2^ = 0.713). The fact that, in the final model, sex, age, income, education, and food security also remain significant indicates the food agency is a comprehensive measure that not only is a proxy for cooking and food skills but also captures differences in attitudes, confidence, perceptions, and structural factors as well as the effect of other socioeconomic factors that influence approaches to food and cooking. The construct of food agency and the CAFPAS measurement tool are therefore a useful lens to understand cooking as a health behavior. In addition, these results provide support for a focus not only on skills but also on self-efficacy/confidence, attitudes, and perceptions of different needs based on structural and economic factors in cooking and diet behavior change interventions. 

The relationship between specific cooking and food skills and dietary intake has not always been entirely clear and consistent, with some studies finding a positive correlation between higher cooking/ food skills and improved dietary intake [6,41] and others painting a more complex picture [11,23]. Cooking and food skills have recently been measured by focusing on a list of specific skills or tasks (e.g., boiling, mixing, sautéing, or microwaving food; reading labels; using grocery lists; etc.) determined *a priori* by the research team [22,42]. Such skills may not always be applicable to everyone in all contexts and may not always be associated with healthier eating (e.g., baking a cake or preparing a gratin) [41]. An additional complicating factor for this approach is the fact that cooking is a highly complex and contextually dependent behavior [14,43], with no set definition [16,44] and no one “right way” to achieve one’s food-related goals, particularly as the act of cooking evolves as the food system and food supply changes [45]. In some contexts, some skills or actions will be relevant, and in other contexts, different strategies may be needed. In addition, self-rated cooking- and food-skill abilities do not measure how often one uses those skills, which also perhaps explains some of the complexity of measuring the relationship between cooking skills and diet quality [42]. The results from the present study demonstrate that, although the CAFPAS does not directly measure predetermined specific skills, food agency is highly correlated with both self-perceived cooking and food skills and with dietary-quality outcomes, while also providing a multidimensional measure of how one approaches food and cooking. This type of more flexible and transferable scale should be examined in other populations and with more comprehensive measures of diet quality to fully understand how food agency functions in different contexts.

In this study, we find that, among both parents and adults, higher food agency is associated with more frequent cooking of breakfast, lunch, and dinner; more frequent cooking from scratch; and less frequent cooking with packaged products. We also find that higher adult food agency is associated with more frequent consumption of fruit and other vegetables and less frequent consumption of fried and other potatoes. Higher parental food agency is associated with less frequent consumption of fried and other potatoes and more frequent consumption of fruit, 100% fruit juice, and other vegetables among 2–9-year-old children. These findings indicate that interventions focusing on increasing food agency may be helpful for increasing cooking frequency, particularly scratch ingredient cooking, and may help improve diet quality and diet-related health outcomes—potentially both for parents as well as for children. 

Cooking frequency is associated with a healthier diet [2,3,4,5,6,46], and strong causal evidence links consumption of packaged, ultra-processed foods with numerous poor health outcomes [8]. Ultra-processed, packaged products [47] are ubiquitous in the food environment in high-income (and increasingly middle- and low-income countries), and recently, cooking at home is recommended by national dietary guidelines and public health practitioners as a means of consuming a healthier and less-processed diet [1,48]. The present findings suggest that policies and interventions to increase food agency may be a key strategy to helping the public cooking more and to cook more from scratch ingredients. In the US, incorporating a food agency approach in SNAP-Ed (the education component of the Supplemental Nutrition Assistant Program (SNAP)) and in the WIC nutrition assistance program and increasing funding for and availability of a modern home economics curriculum (focused on food agency) in K–12 schools, universities, and community colleges are possible policy approaches to increasing food agency. Numerous community cooking interventions already exist that could also incorporate a food agency approach. 

### Limitations

Our findings should be considered in light of several limitations. First, though we did use an online census-matched panel and quotas to achieve a sample that closely reflects the US population, our samples are not truly nationally representative. However, the fact that the demographics of the parent and adult samples do align with the quotas we specified and the fact that, in both samples, estimates of cooking frequency closely mirror estimates from other nationally representative surveys mitigates some of this concern. Second, due to concerns about making the survey too lengthy, we did not use a full food frequency questionnaire or 24-hour dietary recall to measure dietary intake. Therefore, the present results do not shed light on the relationship between food agency and overall diet quality. This is an important area for future research. Third, all measures included in this study are self-reported and may be subject to self-report bias, social-desirability bias, and recall bias. Finally, these data are cross-sectional and we therefore cannot make any causal inferences about the relationship between food agency and cooking skills, food skills, cooking confidence, attitudes, perceptions, cooking behavior, or dietary intake. Future longitudinal or intervention studies will be useful to elucidate the causal nature of some of these relationships. 

## 5. Conclusions

In this study consisting of two large national samples of US adults, we examined factors associated with food agency including various socioeconomic factors; cooking skills; food skills; and cooking related confidence, attitudes, and perceptions. Cooking skills; food skills; and cooking confidence, attitudes, and perceptions explain a large proportion of the variance associated with differences in food agency. We find that food agency is associated with more frequent cooking, more frequent scratch cooking, and less frequent use of packaged products and with healthier dietary intake among both adults and children. The CAFPAS may, therefore, be a useful and concise summary measure to capture the complex behavior of food preparation and procurement. Food agency is an effective lens for understanding cooking and food skills and behaviors and may be useful for policy approaches and interventions to increase cooking frequency and to encourage healthy diets. These findings add to the emerging literature about the role of cooking skills and food skills and the broader construct of food agency for shaping cooking behavior, food choices, and diet quality. 

## Figures and Tables

**Table 1:** Characteristics of the study samples.

	Combined Sample	General Adult Sample	Parent Sample
Total (*n*)	2856	1457	1399
Sex (*n* (%))			
Male	1200 (42.1)	718 (49.3)	482 (34.5)
Female	1654 (58.0)	738 (50.7)	916 (65.5)
Age (mean (SD))	41.1 (13.9)	46.5 (15.8)	35.5 (8.5)
Age (*n* (%))			
18–29	598 (21.0)	251 (17.3)	347 (24.8)
30–44	1308 (45.9)	448 (30.8)	860 (61.5)
45–64	708 (24.9)	527 (36.2)	181 (12.9)
≥65	236 (8.3)	228 (15.7)	8 (0.6)
Race/ethnicity (*n* (%))			
Non-Hispanic White	1327 (46.7)	684 (47.1)	643 (46.2)
Non-Hispanic Black	437 (15.4)	197 (13.6)	240 (17.3)
Hispanic	781 (27.5)	407 (28.1)	374 (26.9)
Other	297 (10.5)	163 (11.2)	134 (9.6)
Household size (*n* (%))			
1–4 people	2206 (77.2)	1295 (88.9)	911 (65.1)
5 or more people	650 (22.8)	162 (11.1)	488 (34.9)
Children < 18 years old in the home (*n* (%))			
Yes	2218 (77.7)	819 (56.2)	1399 (100)
No	638 (22.3)	638 (43.8)	0 (0)
Number of children (mean (SD) )	1.78 (1.43)	1.28 (1.49)	2.30 (1.14)
Ages of children (*n* (%))			
≥1 child aged 0–5 years	1058 (37.0)	161 (11.1)	897 (64.1)
≥1 child aged 6–11 years	1103 (38.6)	196 (13.5)	907 (64.8)
≥1 child aged 12–19 years	1053 (36.9)	635 (43.6)	418 (29.9)
Reference child age (mean (SD) )	N/A	N/A	6.4 (2.3)
Reference child sex (*n* (%))			
Male	N/A	N/A	769 (55.0)
Female	N/A	N/A	630 (45.0)
Marital status (*n* (%))			
Married	1598 (56.2)	693 (47.8)	905 (64.9)
Living with a partner	301 (10.6)	108 (7.5)	193 (13.9)
Divorced/separated/Widowed	313 (11.0)	213 (14.7)	100 (7.2)
Never married	632 (22.2)	436 (30.1)	196 (14.1)
Household income (*n* (%))			
Less than $50,000	1277 (45.4)	610 (42.5)	667 (48.3)
$50,000–$100,000	932 (33.1)	487 (34.0)	445 (32.3)
More than $100,000	605 (21.5)	337 (23.5)	268 (19.4)
Education (*n* (%))			
High school diploma or less	962 (33.8)	485 (33.3)	477 (34.3)
Some college	687 (24.1)	326 (22.4)	361 (25.9)
College or graduate degree	1199 (42.1)	645 (44.3)	554 (39.8)
Employment status (*n* (%))			
Working full time	1334 (47.3)	612 (42.7)	722 (52.0)
Working part time	315 (11.2)	168 (11.7)	147 (10.6)
Not working but looking for work	222 (7.9)	144 (10.0)	78 (5.6)
Not working and not looking for work	412 (14.6)	379 (26.4)	33 (2.4)
Stay at home parent	540 (19.1)	131 (9.1)	409 (29.5)
SNAP ^b^ participation (*n* (%))			
Yes	491 (17.4)	175 (12.1)	316 (23.0)
No	2331 (82.6)	1,270 (87.9)	1061 (77.1)
WIC ^c^ participation (*n* (%))			
Yes	259 (9.1)	36 (2.5)	223 (16.1)
No	2582 (90.9)	1417 (97.5)	1165 (83.9)
Household food insecurity (*n* (%))			
We have enough of the foods we want to eat	1911 (67.4)	1043 (72.2)	868 (62.4)
We have enough but not always the kinds of food we want to eat	722 (25.5)	319 (22.1)	403 (29.0)
Sometimes we do not have enough to eat	171 (6.0)	70 (4.8)	101 (7.3)
We often do not have enough to eat	32 (1.1)	13 (0.9)	19 (1.4)
Weight status ^a^ (*n* (%))			
Healthy	495 (35.2)	495 (35.2)	440 (32.8)
Overweight	489 (34.8)	489 (34.8)	415 (30.9)
Obese	422 (30.0)	422 (30.0)	487 (36.3)

^a^ Based on Body Mass Index (BMI) score. Healthy weight status = 18.5 < BMI < 24.99. Overweight = 25.00 ≤ BMI < 30.00. Obese = BMI ≥ 30. BMI scores were calculated based on self-reported height and weight information. ^b^ Supplemental Nutrition Assistance Program. ^c^ Special Supplemental Nutrition Program for Women, Infants, and Children. Note: Some variables do not sum to the full sample size due to missing data. Some percentages sum to >100 due to rounding.

**Table 2:** Description of the cooking related study variables.

			Combined Sample		General Adult Sample		Parent Sample	
			*n* = 2856		*n* = 1457		*n* = 1399	
Variable	No. Items	Mean score		SD	Mean score	SD	Mean Score	SD
Food Agency								
Full Food Agency scale ^a^	28	13.07		2.41	12.70	2.37	14.31	2.61
Subscale 1: Food Self-efficacy (e.g., (How much do you agree or disagree with…) Before I start cooking, I usually have a mental plan of all the steps I will need to complete)	13	5.49		1.00	5.27	1.00	5.75	1.00
Subscale 2: Food attitudes (e.g., (How much do you agree or disagree with…) For me, cooking is just something to get through as quickly as possible)	10	4.27		1.00	4.03	1.00	5.31	1.16
Subscale 3: Structural barriers (e.g., (How much do you agree or disagree with…) My job responsibilities prevent me from having the time to prepare meals)	5	3.31		1.00	3.39	1.00	3.25	1.00
Skills								
Cooking skills ^b^ (e.g., (Do you/ how good you are at…) blending foods to make them smooth)	14	67.44		21.19	64.65	22.95	70.34	18.80
Food skills ^b^ (e.g., (How good you are at…) planning how much food to buy)	19	102.90		19.82	102.68	19.94	103.12	19.91
Confidence and Attitudes								
Cooking confidence^c^ (e.g., (How confident are you…) cooking from scratch using fresh ingredients)	4	5.42		1.23	5.37	1.28	5.47	1.17
Positive cooking attitudes ^c^ (e.g., (How much do you agree or disagree with…) Cooking is important to me)	7	5.51		1.18	5.39	1.25	5.64	1.01
Negative cooking attitudes ^c^ (e.g., (How much do you agree or disagree with…) Cooking is stressful)	5	3.25		1.32	3.27	1.34	3.24	1.30
Perceptions								
Convenience foods ^c^ (Factor 1) (e.g., (I would say I have cooked if I…) used the oven to heat up store-bought frozen or packaged items such as chicken nuggets, French fries, or fish)	6	4.05		1.55	4.03	1.56	4.08	1.55
Scratch ingredients ^c^ (Factor 2) (e.g., (I would say I have cooked if I…) made something on the stove or oven using mostly scratch or fresh ingredients)	4	5.89		0.91	5.82	0.92	6.00	0.88
Role of heat ^c^ (Factor 3) (e.g., (I would say I have cooked if I…) chopped vegetables to make a salad and used a store-bought salad dressing)	4	4.58		1.38	4.56	1.38	4.61	1.37

^a^ Standardized scale: Each subscale is divided by the standard deviation, and then, subscales scores are summed to create the overall score. ^b^ Additive scale: Responses to each of the items in the set are summed. ^c^ Mean scale: The mean of all items in the set are taken to create an overall mean score.

**Table 3:** Univariate associations with food agency among the general adult population and parents of 2–9-year-old children.

	Combined Sample		General Sample		Parent Sample	
	*n* = 2856		*n* = 1457		*n* = 1399	
	Mean (SE)	*p*-value	Mean (SE)	*p*-value	Mean (SE)	*p*-value
Sex						
Male	12.79 (0.06)	(ref)	12.44 (0.09)	(ref)	14.01 (0.12)	(ref)
Female	13.28 (0.06)	<0.001	12.94 (0.09)	<0.001	14.47 (0.09)	0.002
Age						
18–29	12.73 (0.10)	(ref)	11.77 (0.15)	(ref)	14.43 (0.14)	(ref)
30–45	12.89 (0.07)	0.20	12.46 (0.11)	<0.001	14.12 (0.09)	0.06
45–64	13.57 (0.09)	<0.001	13.17 (0.10)	<0.001	14.95 (0.19)	0.03
≥65	13.42 (0.16)	<0.001	13.05 (0.15)	<0.001	15.44 (0.92)	0.28
Race/ethnicity						
NH White	13.02 (0.07)	(ref)	12.76 (0.09)	(ref)	14.12 (0.10)	(ref)
NH Black	13.45 (0.12)	0.001	12.87 (0.16)	0.56	14.88 (0.17)	<0.001
Hispanic	13.05 (0.09)	0.822	12.59 (0.12)	0.23	14.39 (0.13)	0.11
Other	12.83 (0.14)	0.203	12.47 (0.19)	0.16	14.03 (0.22)	0.72
Parents of children 0–18 in the home						
Yes	13.17 (0.05)	(ref)	12.94 (0.08)	(ref)	n/a	n/a
No	12.73 (0.10)	<0.001	12.38 (0.09)	<0.001	n/a	n/a
Household income						
Less than $50,000	13.11 (0.07)	(ref)	12.73 (0.10)	(ref)	14.35 (0.10)	(ref)
$50,000–$100,000	13.08 (0.08)	0.763	12.66 (0.11)	0.65	14.37 (0.12)	0.90
More than $100,000	13.00 (0.10)	0.261	12.69 (0.13)	0.80	14.09 (0.16)	0.16
Education						
High school diploma or less	13.17 (0.08)	(ref)	12.65 (0.11)	(ref)	14.58 (0.12)	(ref)
Some college	13.23 (0.09)	0.602	12.94 (0.13)	0.09	14.39 (0.14)	0.31
College or graduate degree	12.90 (0.07)	0.012	12.61 (0.09)	0.77	14.04 (0.11)	0.001
Employment status						
Working full time	12.72 (0.06)	(ref)	12.42 (0.10)	(ref)	13.88 (0.09)	(ref)
Working part time	12.85 (0.13)	0.404	12.48 (0.18)	0.78	14.07 (0.21)	0.43
Not working but looking for work	12.91 (0.16)	0.269	12.52 (0.20)	0.65	14.22 (0.29)	0.26
Not working and not looking for work	13.53 (0.12)	<0.001	13.11 (0.12)	<0.001	15.55 (0.44)	<0.001
Stay at home parent	13.79 (0.10)	<0.001	13.27 (0.21)	<0.001	15.09 (0.13)	<0.001
Household food insecurity						
We have enough of the foods we want to eat	13.28 (0.05)	(ref)	12.87 (0.07)	(ref)	14.58 (0.09)	(ref)
We have enough but not always the kinds of food we want to eat	12.67 (0.09)	<0.001	12.26 (0.13)	<0.001	13.91 (0.13)	<0.001
Sometimes we do not have enough to eat	12.67 (0.18)	0.002	12.56 (0.28)	0.28	13.69 (0.26)	0.001
We often do not have enough to eat	12.45 (0.42)	0.052	11.11 (0.65)	0.01	14.34 (0.59)	0.69

Note: “Ref” stands for reference group.

**Table 4:** Hierarchical regression results predicting food agency among the combined sample of the general adult population and parents of 2–9-year-old children (*n* = 2856).

	Model 1 β (SE)		Model 2 β (SE)		Model 3 β (SE)		Model 4 β (SE)	
Sex								
Male	(ref)		(ref)		(ref)		(ref)	
Female	0.55 ***	(0.09)	0.55 ***	(0.09)	0.07	(0.07)	0.18 ***	(0.05)
Age	0.03 ***	(0.00)	0.03 ***	(0.00)	0.03 ***	(0.00)	0.03 ***	(0.00)
Race/ethnicity								
NH White	(ref)		(ref)		(ref)		(ref)	
NH Black	0.43 **	(0.13)	0.39 **	(0.13)	0.26 *	(0.11)	0.12	(0.07)
Hispanic	0.13	(0.11)	0.12	(0.11)	0.07	(0.09)	−0.03	(0.06)
Other	−0.14	(0.15)	−0.10	(0.16)	−0.09	(0.12)	−0.14	(0.09)
Number of children in household	0.12 ***	(0.03)	0.13 ***	(0.03)	0.04	(0.03)	0.02	(0.02)
Household income								
Less than $50,000			(ref)		(ref)		(ref)	
$50,000–$100,000			−0.16	(0.11)	−0.22 *	(0.08)	−0.18 **	(0.06)
More than $100,000			−0.38 **	(0.13)	−0.40 ***	(0.10)	−0.33 ***	(0.07)
Education								
High school diploma or less			(ref)		(ref)		(ref)	
Some college			−0.03	(0.12)	−0.25 **	(0.10)	−0.16 *	(0.07)
College or graduate degree			−0.34 **	(0.11)	−0.46 ***	(0.09)	−0.23 ***	(0.06)
Household food insecurity								
We have enough of the foods we want to eat			(ref)		(ref)		(ref)	
We have enough but not always the kinds of food we want to eat			−0.68 ***	(0.11)	−0.36 ***	(0.09)	−0.17 **	(0.06)
Sometimes we do not have enough to eat			−0.83 ***	(0.20)	−0.49 **	(0.16)	−0.08	(0.11)
We often do not have enough to eat			−1.06 *	(0.42)	−0.60	(0.33)	0.09	(0.23)
Cooking Skills					0.04 ***	(0.00)	0.01 ***	(0.00)
Food skills					0.04 ***	(0.00)	0.01 ***	(0.00)
Cooking confidence							0.15 ***	(0.04)
Positive attitudes							0.65 ***	(0.04)
Negative Attitudes							−0.73 ***	(0.02)
Cooking Perceptions								
F1: Convenience foods							−0.12 ***	(0.02)
F2: Scratch ingredients							0.19 ***	(0.03)
F3: Heat							−0.11 ***	(0.02)
Model R^2^	0.044		0.064		0.415		0.713	
Change in R^2^ from prior model	n/a		0.021, *p* < 0.001		0.351, *p* < 0.001		0.298, *p* < 0.001	

Note: *** *p* < 0.001, ** *p* < 0.01, * *p* < 0.05. “Ref” stands for reference group.

## Supplementary Materials

The following are available online at https://www.mdpi.com/2072-6643/12/3/877/s1, Table S1: Hierarchical regression results predicting food agency among the general adult population, Table S2: Hierarchical regression results predicting food agency among parents of 2–9-year-old children, Figure S1: Associations between food agency and cooking behavior among the combined sample (*n* = 2856).
