# Supplementary material for: Food Agency in the United States: Associations with Cooking Behavior and Dietary Intake
Source: Nutrients. 2020 Mar 24;12(3):877. doi: 10.3390/nu12030877 (PMC7146410; doi:10.3390/nu12030877)
Supplement: Supplementary file 1 [file nutrients-12-00877-s001.zip › nutrients-751957-SI.docx]

**Table S1:** Hierarchical regression results predicting food agency among the general adult population.

|  | **Model 1**  **β (SE)** | | **Model 2**  **β (SE)** | | **Model 3**  **β (SE)** | | **Model 4**  **β (SE)** | |
| --- | --- | --- | --- | --- | --- | --- | --- | --- |
|  |  |  |  |  |  |  |  |  |
| Sex |  |  |  |  |  |  |  |  |
| Male | [ref] |  | [ref] |  | [ref] |  | [ref] |  |
| Female | 0.59*** | (0.12) | 0.64*** | (0.12) | 0.11 | (0.10) | 0.17* | (0.07) |
| Age | 0.03*** | (0.00) | 0.03*** | (0.00) | 0.03*** | (0.00) | 0.03*** | (0.00) |
| Race/ethnicity |  |  |  |  |  |  |  |  |
| NH White | [ref] |  | [ref] |  | [ref] |  | [ref] |  |
| NH Black | 0.10 | (0.19) | 0.03 | (0.19) | -0.04 | (0.15) | -0.00 | (0.11) |
| Hispanic | 0.03 | (0.15) | -0.01 | (0.15) | -0.01 | (0.12) | -0.11 | (0.08) |
| Other | -0.19 | (0.20) | -0.18 | (0.21) | -0.07 | (0.16) | -0.19 | (0.12) |
| Number of children in household | 0.11** | (0.04) | 0.12** | (0.04) | 0.05 | (0.03) | 0.02 | (0.02) |
| Household income |  |  |  |  |  |  |  |  |
| Less than $50,000 |  |  | [ref] |  | [ref] |  | [ref] |  |
| $50,000-$100,000 |  |  | -0.18 | (0.15) | -0.24* | (0.12) | -0.19* | (0.08) |
| More than $100,000 |  |  | -0.29 | (0.17) | -0.36** | (0.14) | -0.27** | (0.09) |
| Education |  |  |  |  |  |  |  |  |
| High school diploma or less |  |  | [ref] |  | [ref] |  | [ref] |  |
| Some college |  |  | 0.18 | (0.17) | -0.04 | (0.13) | -0.06 | (0.09) |
| College or graduate degree |  |  | -0.13 | (0.15) | -0.25* | (0.12) | -0.11 | (0.08) |
| Household food insecurity |  |  |  |  |  |  |  |  |
| We have enough of the foods we want to eat |  |  | [ref] |  | [ref] |  | [ref] |  |
| We have enough, but not always the kinds of food we want to eat |  |  | -0.64*** | (0.16) | -0.39** | (0.12) | -0.20* | (0.09) |
| Sometimes we do not have enough to eat |  |  | -0.45 | (0.30) | -0.37 | (0.24) | -0.16 | (0.17) |
| We often do not have enough to eat |  |  | -1.93** | (0.64) | -1.67** | (0.51) | -0.68 | (0.36) |
| Cooking Skills |  |  |  |  | 0.03*** | (0.00) | 0.01* | (0.00) |
| Food skills |  |  |  |  | 0.04*** | (0.00) | 0.01*** | (0.00) |
| Cooking confidence |  |  |  |  |  |  | 0.20*** | (0.05) |
| Positive attitudes |  |  |  |  |  |  | 0.54*** | (0.05) |
| Negative Attitudes |  |  |  |  |  |  | -0.73*** | (0.03) |
| Cooking Perceptions |  |  |  |  |  |  |  |  |
| F1: Convenience foods |  |  |  |  |  |  | -0.15*** | (0.03) |
| F2: Scratch ingredients |  |  |  |  |  |  | 0.17*** | (0.04) |
| F3: Heat |  |  |  |  |  |  | -0.10** | (0.03) |
| Model R^2^ | 0.067 |  | 0.086 |  | 0.427 |  | 0.724 |  |
| Change in R^2^ from prior model | n/a | | 0.019, *p* < 0.001 | | 0.341, *p* < 0.001 | | 0.297, *p* < 0.001 | |

Note: *** *p* < 0.001, ** *p* < 0.01, * *p* < 0.05.

**Table S2:** Hierarchical regression results predicting food agency among parents of 2–9 year-old children.

|  | **Model 1**  **β (SE)** | | **Model 2**  **β (SE)** | | **Model 3**  **β (SE)** | | **Model 4**  **β (SE)** | |
| --- | --- | --- | --- | --- | --- | --- | --- | --- |
|  |  |  |  |  |  |  |  |  |
| Sex |  |  |  |  |  |  |  |  |
| Male | [ref] |  | [ref] |  | [ref] |  | [ref] |  |
| Female | 0.47** | (0.15) | 0.42** | (0.15) | 0.00 | (0.12) | 0.16 | (0.08) |
| Age | 0.03** | (0.01) | 0.03** | (0.01) | 0.02** | (0.01) | 0.03*** | (0.01) |
| Race/ethnicity |  |  |  |  |  |  |  |  |
| NH White | [ref] |  | [ref] |  | [ref] |  | [ref] |  |
| NH Black | 0.77*** | (0.20) | 0.78*** | (0.20) | 0.55*** | (0.16) | 0.25* | (0.11) |
| Hispanic | 0.30 | (0.17) | 0.34 | (0.17) | 0.19 | (0.14) | 0.06 | (0.10) |
| Other | -0.05 | (0.25) | 0.02 | (0.26) | -0.14 | (0.20) | -0.12 | (0.14) |
| Number of children in household | 0.10 | (0.07) | 0.08 | (0.07) | 0.03 | (0.06) | 0.03 | (0.04) |
| Ages of children |  |  |  |  |  |  |  |  |
| >1 children aged 0-5 years | [ref] |  | [ref] |  | [ref] |  | [ref] |  |
| >1 children aged 6-11 years | -0.30 | (0.18) | -0.24 | (0.18) | -0.27 | (0.14) | -0.07 | (0.10) |
| >1 children aged 12-19 years | -0.22 | (0.24) | -0.14 | (0.24) | -0.29 | (0.19) | -0.03 | (0.13) |
| Household income |  |  |  |  |  |  |  |  |
| Less than $50,000 |  |  | [ref] |  | [ref] |  | [ref] |  |
| $50,000-$100,000 |  |  | -0.13 | (0.17) | -0.16 | (0.13) | -0.17 | (0.09) |
| More than $100,000 |  |  | -0.48* | (0.21) | -0.39* | (0.16) | -0.38*** | (0.11) |
| Education |  |  |  |  |  |  |  |  |
| High school diploma or less |  |  | [ref] |  | [ref] |  | [ref] |  |
| Some college |  |  | -0.32 | (0.19) | -0.50*** | (0.15) | -0.28** | (0.10) |
| College or graduate degree |  |  | -0.62*** | (0.17) | -0.74*** | (0.14) | -0.37*** | (0.10) |
| Household food insecurity |  |  |  |  |  |  |  |  |
| We have enough of the foods we want to eat |  |  | [ref] |  | [ref] |  | [ref] |  |
| We have enough, but not always the kinds of food we want to eat |  |  | -0.77*** | (0.16) | -0.31* | (0.13) | -0.13 | (0.09) |
| Sometimes we do not have enough to eat |  |  | -1.19*** | (0.28) | -0.59** | (0.22) | 0.02 | (0.16) |
| We often do not have enough to eat |  |  | -0.55 | (0.60) | 0.11 | (0.48) | 0.64 | (0.33) |
| Cooking Skills |  |  |  |  | 0.05*** | (0.00) | 0.01*** | (0.00) |
| Food skills |  |  |  |  | 0.04*** | (0.00) | 0.01*** | (0.00) |
| Cooking confidence |  |  |  |  |  |  | 0.10 | (0.05) |
| Positive attitudes |  |  |  |  |  |  | 0.87*** | (0.06) |
| Negative Attitudes |  |  |  |  |  |  | -0.77*** | (0.04) |
| Cooking Perceptions |  |  |  |  |  |  |  |  |
| F1: Convenience foods |  |  |  |  |  |  | -0.09** | (0.03) |
| F2: Scratch ingredients |  |  |  |  |  |  | 0.22*** | (0.05) |
| F3: Heat |  |  |  |  |  |  | -0.13*** | (0.04) |
| Model R^2^ | 0.027 |  | 0.059 |  | 0.417 |  | 0.719 |  |
| Change in R^2^ from prior model | n/a | | 0.031, *p* < 0.001 | | 0.359, *p* < 0.001 | | 0.302, *p* < 0.001 | |

Note: *** *p* < 0.001, ** *p* < 0.01, * *p* < 0.05.

**Figure S1.** Associations between food agency and cooking behavior among the combined sample (*n* = 2,856). Note: Based on poisson regressions adjusted for sex, age, race/ethnicity, number of children, income, education, food security status, and cooking perceptions. Frequency of cooking behaviors is measured over the past 7 days. Difference from Q1 significant at: *** *p* < 0.001, ** *p* < 0.01, * *p* < 0.05.
